# Perception of alcohol policies by consumers of unrecorded alcohol - an exploratory qualitative interview study with patients of alcohol treatment facilities in Russia

**DOI:** 10.1186/s13011-019-0234-1

**Published:** 2019-11-21

**Authors:** Maria Neufeld, Hans-Ulrich Wittchen, Lori E. Ross, Carina Ferreira-Borges, Jürgen Rehm

**Affiliations:** 1 Institute for Clinical Psychology and Psychotherapy, TU Dresden, Chemnitzer Str. 46, 01187 Dresden, Germany; 2 WHO European Office for Prevention and Control of Noncommunicable Diseases, Moscow, Leontyevsky Pereulok 9, Moscow, Russian Federation 125009; 3 Institute for Mental Health Policy Research, Centre for Addiction and Mental Health (CAMH), 33 Russell Street, Toronto, ON M5S 2S1 Canada; 4 Department of Psychiatry, University of Toronto, 250 College Street, 8th Floor, Toronto, ON M5T 1R8 Canada; 5 Dalla Lana School of Public Health, University of Toronto, 155 College Street, 6th Floor, Toronto, ON M5T 3M7 Canada; 6 Campbell Family Mental Health Research Institute, CAMH, 250 College Street, Toronto, ON M5T 1R8 Canada; 7 Institute of Medical Science (IMS), University of Toronto, Medical Sciences Building, 1 King’s College Circle, Room 2374, Toronto, ON M5S 1A8 Canada; 8 Department of International Health Projects, Institute for Leadership and Health Management, I.M. Sechenov First Moscow State Medical University, Trubetskaya str., 8, b. 2, Moscow, Russian Federation 119992

**Keywords:** Russia, Qualitative interviews, Unrecorded alcohol, Alcohol use disorders, Alcohol policy, Alcohol availability, Alcohol taxation, Surrogate alcohol

## Abstract

**Background:**

Over the last decade Russia has introduced various policy measures to reduce alcohol consumption and alcohol-related harm at the population level. Several of these policies, such as higher pricing and taxation or restrictions of availability, may not work in the case of unrecorded alcohol consumption; they may encourage consumers to switch to unrecorded alcohol and even increase consumption. In the present qualitative interview study we explore the perception of the recently implemented alcohol policies by patients diagnosed with alcohol dependence in two Russian cities in the years 2013–2014 and shed light on possible entry-points to prevention.

**Methods:**

Semi-structured in-depth expert interviews were conducted with 25 patients of state-run drug and alcohol treatment centers in two Russian cities in 2013 and 2014. The interviews were analyzed using thematic content analysis**.**

**Results:**

All of the interviewed participants have consumed unrecorded at some point with the majority being regular consumers, mostly switching between recorded and unrecorded alcohol depending on the situation, as predominantly defined by available money and available sources of alcohol. Low price and high availability were reported as the main reasons for unrecorded consumption. Participants voiced a general mistrust of the recently implemented alcohol regulations and viewed them largely as ineffective. They expressed particular concerns over price increases and restriction of night sales of alcoholic beverages. Substantial shifts within the unrecorded alcohol market were reported, with a decreasing availability of home-made beverages in favor of alcohol surrogates in the form of non-beverage alcohol, medicinal and cosmetic compounds. At the same time consumption of home-made alcoholic beverages was seen as a strategy to avoid counterfeit alcohol, which was frequently reported for retail sale.

**Conclusions:**

Despite the alcohol policy changes in the last years in Russia, consumption of unrecorded alcohol remained common for people with alcohol dependence. Reduction of availability of unrecorded alcohol, first and foremost in the form of cheap surrogates, is urgently needed to reduce alcohol-related harm. Implementation of screening and brief interventions for excessive alcohol consumption in various contexts such as primary healthcare settings, trauma treatment services or the workplace could be another important measure targeting consumers of unrecorded alcohol.

## Background

### Unrecorded alcohol consumption and associated harm

Russia has one of the highest levels of per capita alcohol consumption in the world with prevailing risky patterns of drinking [46], resulting in one of the highest levels of alcohol-attributable mortality and harm globally [15, 37].

In the current literature, consumption of unrecorded alcohol (alcohol, which is consumed as a beverage, but is not accounted for in any official statistics such as statistics on alcohol taxation and/or sales) is broadly discussed as one of the key factors for the alcohol-attributable health detriments observed in Russia [15, 40, 41]. Drinking alcohol surrogates, a particularly cheap subgroup of unrecorded alcohol consisting of liquids that contain alcohol but are officially not intended for human consumption, has been persistently linked to extreme binge drinking patterns, alcohol use disorders (AUDs) [3, 18, 19, 23], and other alcohol-related behaviors predictive of poor health and mortality [13, 36, 42]. Since most of the surrogates found in Russia contain a high concentration of ethanol (60% and above), they provide not only a cheap, but also a concentrated source of alcohol and are therefore popular with the poorest population segments who seek high levels of blood alcohol concentration [31]. Although some toxicological studies report the presence of toxic compounds in some types of unrecorded alcohol products found in Russia [15, 18, 40], the most recent meta-analysis of unrecorded alcohol consumption and harm suggests that the main harm stems from ethanol itself – be it in Russia or worldwide [31].

However, in December 2016/January 2017 more than 100 individuals were poisoned in the Siberian city of Irkutsk after consuming a methanol-based counterfeit bath lotion, which was misused as surrogate alcohol by the consumers and mislabeled as containing ethanol by the producers. As a result, 74 people died of methanol poisoning, most of whom were middle-aged individuals from lower socio-economic strata living in a working class district of the city [21, 22]. According to official statistics of the Russian Federal Service for Surveillance on Consumer Rights Protection and Human Wellbeing each year about 1000 methanol poisonings are registered, of which almost 800 result in fatalities [34].

The problem of unrecorded alcohol consumption and associated harms is, however, not unique to Russia. Consumption of this type of alcohol is observed globally, although large variations exist between geographical areas and income groups, raising the most concern in low and lower middle income countries [26]. The presented results of this study may be partly transferrable to other geographical regions where consumption of unrecorded alcohol also represents a challenge for public health and policy (for instance the neighboring country Belarus) and may be useful for developing specific prevention measures especially in those areas, where this phenomenon remains poorly explored.

### Alcohol control policies in Russia

Since 2005/2006, Russia has adopted a series of major amendments to its federal alcohol control law and implemented a variety of measures to reduce alcohol consumption and alcohol-related harms at the population level [9, 14, 17, 20, 21]. The main changes concerned stricter requirements and monitoring of the production and distribution chain of alcoholic products, stricter rules for the production of denatured alcohol (alcohol to which certain additives have been added to discourage consumption as beverage alcohol) and the introduction of new excise stamps to curb counterfeiting. One of the key changes was the introduction of the Unified State Automated Information System (EGAIS), which strarting from 2006 collected data on the volumes of produced alcohol and later on important and wholesale and retail sales of alcoholic beverages [21]. The system allowed for more state control over the production and distribution chain of alcohol and aimed at reducing illicit alcohol production in the first place.

Since 2010–2012 higher alcohol pricing and taxation, restrictions of sale locations and sale times (including the introduction of a nation-wide ban on night sales from 11 PM to 8 AM) were enacted in addition to the restrictions on alcohol consumption in public places and certain facilities. The prohibition of internet trade of alcoholic beverages and stricter penalties for unlicensed production, distribution and sale of alcoholic products were introduced as specific measures to fight alcohol counterfeits and illegally produced alcohol [19, 23]. Moreover, a series of decrees temporary banning the sale of non-beverage alcoholic products with an alcohol content above 28% was issued in response to the Irkutsk mass methanol poisoning in 2016–2017 [22], which were turned into a permanent ban in 2018 [24]. Moreover, in 2017 two new sections were introduced to the Criminal Code of the Russian Federation, imposing harsher penalties on illicit production and sale of illicit alcohol [21].

For a general overview of the introduced alcohol policies see: [9, 14, 16, 20]; for an overview of policy changes to reduce harm from unrecorded alcohol: [21].

### Potential impact of alcohol policies on consumption of unrecorded alcohol

Research suggests that these policy changes have had success in reducing alcohol-related mortality, or were at least coinciding with a major decline in mortality over the last decade [7–10, 14, 16, 17, 20, 25, 27, 28, 39].

For the consumption of unrecorded alcohol, however, it is difficult to assess what impact the usual measures like pricing or restrictions on availability of recorded alcohol have had, due to the concealed nature of the unrecorded market. Similarly, there is only a limited number of studies, focusing on drinking patterns of unrecorded alcohol and appropriate measures to tackle the harms of this type of alcohol [3, 23, 43].

Consumption of unrecorded alcohol is highly relevant for public health, since AUDs are one of the most common types of mental disorders and are associated with substantial burden of disease and economic costs [32, 46]. Moreover, the heavy drinkers in a population are also the ones who consume proportionally the largest amounts of available alcohol. According to the Russian Longitudinal Monitoring Survey [33], in 2013 and 2014 (the years of the study), 1.3% of respondents could be classified as (chronic) heavy drinkers based on the past month drinking habits (at least 40 g/day for females and 60 g/day for males, according to the common definition to define heavy drinker as defined by the European Medicines Agency or the WHO). These persons drank 30.7 and 32.2% of the total reported alcohol in this sample in 2013 and 2014, respectively. Although consumption of surrogate alcohol was not fully covered by the survey, the data demonstrated that heavy drinking correlated with unrecorded alcohol consumption and purchase, which might include surrogates as well [12].

Previous studies exploring the phenomenon of unrecorded consumption in Russia using individual-based data concluded that consumption of highly concentrated unrecorded products is particularly common among individuls residing in rural areas as well as people, who exhibit clinical symptoms of AUD or/and have an established diagnosis, pointing to the socio-economic dimension of the phenomenon and possible implications of and for alcohol control policy. For instance, a study conducted in three rural towns in Russia in 2001 found widespread consumption and production of home-distilled spirits (“samogon”) in the population as well as high frequency of alcohol use, especially among older study participants, who tended to drink more samogon as compared with younger age groups [47]. The author concluded that the loosening of state control over samogon distilling that happened after the dissolution of the Soviet Union has led to the rise in samogon production and that the availability of cheap samogon in economically underdeveloped rural areas poses a serious public health concern. An interview study conducted in 2006–2007 with clients admitted to a clinic for drinking-related problems in the Russian city of Novosibirsk [3] reported that out of all 40 study participants, every participant reported to have consumed surrogate alcohol. High affordability and physical availability of surrogates combined with clinical features of alcohol use disorders such as withdrawal symptoms and craving were named as the main reasons for surrogate consumption. These findings were in line with the results of our own study on drinking patterns of unrecorded alcohol in Russian patients of alcohol-treatment services [23] as well as with similar studies from Belarus [29, 30]. The research outlined points to the complex relationship between socio-economic factors, alcohol use disorders and the availability and affordability of recorded alcohol in the local settings as there is a risk that alcohol control policies such as higher pricing of recorded alcohol might increase surrogate drinking [3], particularly among the most marginalized consumers. The present contribuition therefore aims at exploring these dimensions in greater detail, highlighting how alcohol control policies are perceived by this vulnerable population of alcohol consumers. Itis based on a larger interview study that was conducted in Russian state-run addiction treatment clinics, which covers a broad range of topics related to unrecorded consumption and explores the multidimensional phenomenon of unrecorded alcohol consumption as an entity in itself. While self-reported drinking behaviors and subjective harm of unrecorded alcohol are analyzed in a separate sub-study [23], the present contribution highlights the perceived impact of Russian alcohol policies on consumers of unrecorded alcohol in the years 2013 and 2014. It focuses specifically on the perspective of people diagnosed with alcohol dependence on the different policies and changing alcohol markets in the course of the last decade. Taking the perspective of this special population into account might not only improve the understanding of unrecorded consumption as a phenomenon, but also help to develop specific and adequate policies to reduce harm. Therefore, the main purpose of the study is to understand whether the existing alcohol control policies address unrecorded alcohol consumers in an appropriate manner as well as to identify possible gaps and flaws in regard to the existing measures.

## Method

### Study setting

We expected to find consumers of unrecorded alcohol in a population of patients, who are treated for alcohol dependence in state-run addiction treatment services (in Russian: “narcological” services). Specialized treatment in the Russian narcology system is free of charge, but requires an official registration as a narcological patient, mandating long-term monitoring in an ambulatory setting. The registration limits employment possibilities, is generally seen as stigmatizing and is therefore usually avoided by patients as long possible [2].

Accordingly, our underlying assumption was that patients of state-run narcological clinics are more likely to be of lower socio-economic strata and therefore to consume unrecorded alcohol more likely than patients receiving anonymous treatment in private facilities and treatment centers. Therefore, the study was conducted in an outpatient narcological treatment facility in the city of Barnaul (Siberia) and an inpatient clinic in the city of Petrozavodsk (North-Western region). The two cities were chosen to account for the experiences of unrecorded consumers living in Russia’s periphery. Further details on the study setting are presented in the Additional file 1: Table S1 and S2).

Semi-structured, in-depth, qualitative interviews were chosen to explore the phenomenon of unrecorded alcohol consumption within Russian communities, since we wished to understand the perspectives of consumers of unrecorded alcohol as “experts” [4, 44] on the consequences of the described changes in alcohol policy, including potential unwanted effects of the implemented measures such as price increases.

### Data collection

A total of 25 interviews were conducted; 18 interviews were carried out in an ambulatory clinic in Barnaul in August 2013 and 2014, and another 7 interviews were conducted in an in-patient clinic in Petrozavodsk in September 2014.

All patients diagnosed with alcohol dependence (established diagnosis F10.2) present during the data collection period who agreed to the interview were included in the sample, which resulted in a census sample for the assessment days; in the Barnaul ambulatory center interviews were conducted on the 18th and 20th of August 2013 and the 17th of August 2014, and in the Petrozavodsk in-patient clinic on the 4th of September 2014. Two of the approached patients (one from Barnaul and one from Petrozavodsk) refused to participate in the interview as they reported not to feel well. All patients were recruited in the waiting room of the ambulatory center and the community room of the in-patient clinic by the first author, who had no prior relationship to the patients and presented herself as a researcher according to a prepared script. The participants signed a consent form, where it was explained that the anonymous interview would be conducted for scientific purposes only and would not have any personal consequences with respect to health care provision or other social sanctions. Participants received no compensation for their participation.

The interviews followed a semi-structured interview guide, which was developed on the basis of existing literature on this topic [3, 36] and outcomes of a pilot phase (described below). The open-ended questions of the interview guide probed the following subject areas: perceptions and consumption of different types of unrecorded alcohol, quality of the products, personal drinking patterns and typical drinking scenarios, alcohol-induced harm, price and availability of unrecorded products, perception and impact of alcohol policies and observed changed in the communities. Due to the exploratory nature of the study, the interview guide was flexibly applied in order to permit discussion of other relevant topics raised by participants. The comprehensibility of the interview guide was tested during a pilot phase conducted at a health and recreation resort in a Siberian village not far from Barnaul prior to initiation of formal data collection, encompassing a total of 10 pilot interviews.

The interviews were conducted face to face in Russian, in a private room and recorded by the first author. The average length of the interviews was 28 min, ranging from 11 min to 1 h 51 min. General field-notes were taken after each interview.

### Analysis

The recordings were saved and documented by an identification number, separately from the signed consent forms. The interviews were transcribed and translated into English by the first author, and two random samples of interview transcripts were given to two native Russian speakers, who independently checked for the accuracy of translation against the original sound files. The material was edited to remove any unwanted artefacts, such as “ums” and “ahs” and the translators discussed discrepancies and tried to find adequate translations for Russian slang words to keep as close as possible to the original material. Field notes were typed and used to contextualize the interview setting for each participant.

Open line-by-line coding of the transcripts was performed by the first author using the software program Nvivo. The last author cross-coded a sub-set of transcripts and differences were thoroughly discussed. The overall coding network was developed through repeated careful readings, cross-checking and discussions among the authors, incorporating the initial categories from the interview guide and the pilot study as well as emerging concepts from the study material. All the authors re-read the material to create a thematic map [5] of the entire study. Broader key themes that emerged from the data were: 1) types of unrecorded products, associated quality and subjective harm 2) drinking patterns/scenarios and their social settings 3) social markers of status within communities 4) determinants of production and consumption of unrecorded alcohol 5) impact of alcohol regulations on the consumers and their communities 6) observed changes in communities and shifts in alcohol markets (Additional file 1: Figure S1).

**Fig. 1:**
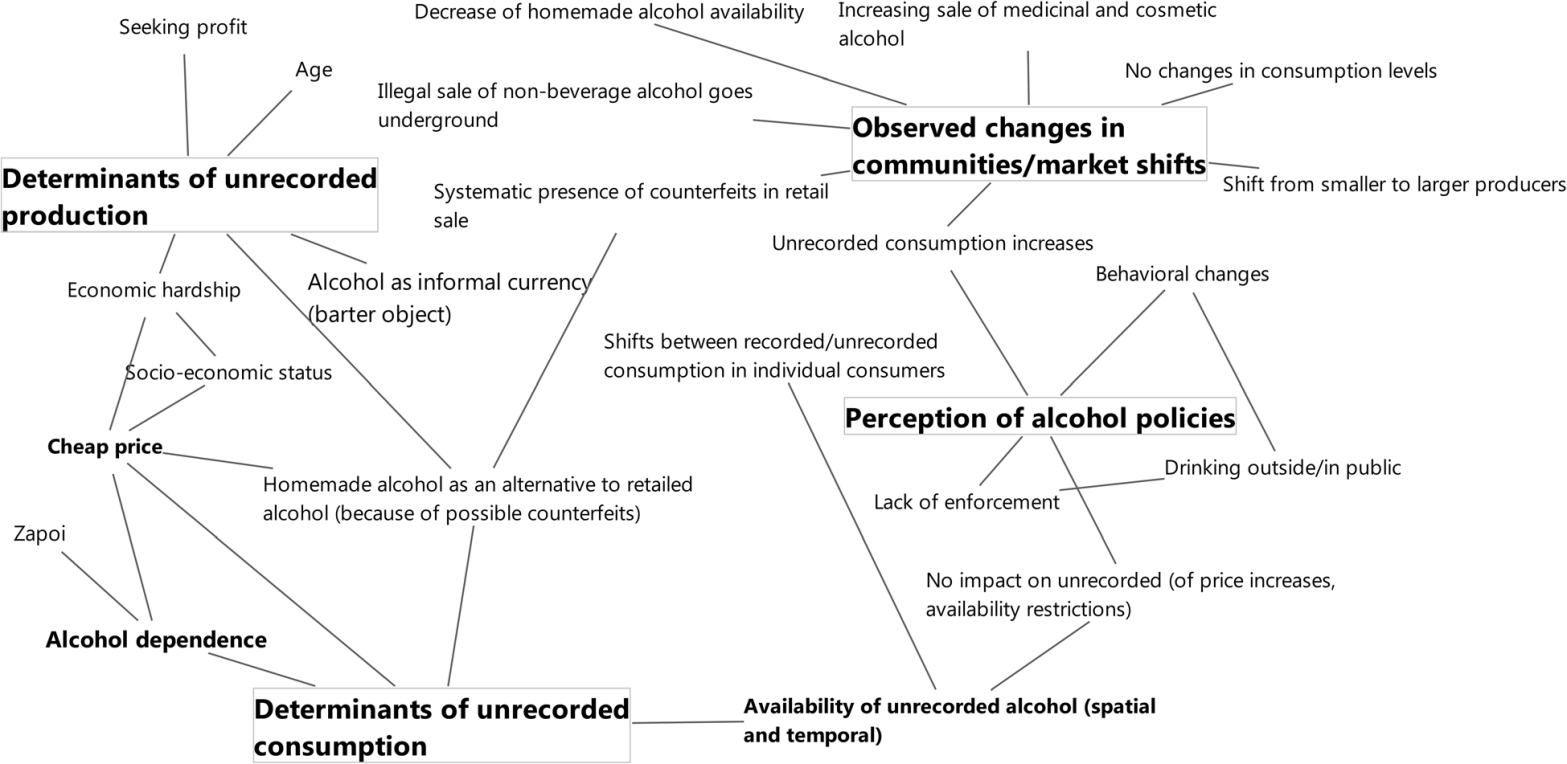
Thematic map of the key themes that emerged from the interview material of the present study

For the present contribution a thematic analysis was performed for the latter three key themes of the study (Fig. 1).

## Results

### Description of the sample

The sample consisted of 17 males and 8 females, the majority of whom lived with their families in the cities (4 lived alone, 4 lived with their partner, 4 lived with their children, 9 lived with their partner and children, 4 lived in an intergenerational family with their children, parents, siblings and/or other relatives). The average age of the interviewees was 39 years (range of 24–78). Most of the participants were either unemployed at the time of the interview or informally employed as unskilled/occasional workers (36 and 28% of the sample size, respectively). Almost two-thirds (64%) reported various periods of unemployment, lasting from weeks to several years, and 2 persons reported imprisonment in their lifetime.

The reported average monthly per capita household income of the sample was 11,841 rubles (332$), with a median of 10,000 rubles (280$), which is almost 2.5 times below the Russian average per capita income of urban households in 2014 [6]. For a more detailed sample description see Additional file 1: Table S3 and Figure S2.

Unrecorded alcohol consumption was pictured as a common und mundane everyday phenomenon in both communities. All interviewees reported to have consumed unrecorded at least at some point in their lives as well as to know other consumers in their surroundings. The majority of the interviewed were regular consumers, mostly switching between recorded and unrecorded alcohol, depending on the situation and the available resources.

### Price and availability of alcohol as the main motives for unrecorded consumption

Home-distilled spirits (“samogon”), counterfeit alcoholic beverages and surrogates, i.e., illegally sold non-beverage alcohol and alcohol-based cosmetic lotions and medicinal compounds from legal retail sale, were all used by our participants. The latter were reported to be the cheapest alcoholic products, affordable even for the poorest population groups, and mentioned in connection with severe forms of alcohol dependence, lower socio-economic status and homelessness of their consumers.

Low price was indicated as the most important reason for consumption of surrogates. Unrecorded alcoholic products - with the exception of certain types of homemade beverages- were mostly consumed when interview participants were experiencing financial difficulties, but were in need of alcohol due to the nature of their AUD. Problematic drinking behaviors like zapoi (continuous drunkenness lasting longer than 2 days) and hang-over drinking (drinking little amounts of alcohol to ease withdrawal symptoms) were frequently mentioned as typical drinking scenarios.

Interview_B13_6 (male, 33 years old):*“Because one has no money. So one has to buy this non-beverage alcohol. Half of the city is drinking it now. For easing the hangover. Or simply if you don’t have enough money to buy something to drink. Even if one has money for a bottle, but you are five people. So you can buy those flacons and it will be more.”*

However, most of the interviewees tended to switch back to recorded alcohol once they had money again, since the consumption of unrecorded alcohol and most importantly surrogates was seen as a marker for social degradation. Other factors, like social norms and relations within a drinking group or drinking environment also influenced the specific beverage choices.

Spatial and temporal availability of alcohol was indicated as the second main reason for unrecorded consumption.

Interview_B13_5 (male, 37 years old): *“It’s not just because of the money, it’s also simply because you cannot buy anything anywhere at night. Only in the one spot you know. Or you go to the pharmacy. The 24 hours one. There is nowhere else, where to buy it.”*

In Barnaul, participants reported that after 9 PM no more alcohol could be legally obtained in the stores, while for Petrozavodsk this occurred after 11 PM, in line with the regional implementation of the Federal Law. Limited sale hours of alcohol were frequently described as a strong factor influencing the situation of people with alcohol dependence, regardless of whether they were consuming unrecorded alcohol or recorded beverages.

Interview_B13_2 (female, 36 years old): “*On other occasions, if I have money, I just go and buy normal beer and that’s all. And I eat and everything is all right then. First, it depends on the money. And at night time you can’t buy anywhere. Only in a bar. [Did something like that happen? You went to a bar?] No, I didn’t go to a bar. [So you just buy a lot in advance? Or you try to endure?] No. Yes, or I take a sleeping pill. Or I suffer. Just go in rounds in my flat and wait.”*

Naturally, the time limitations did not apply for the sale of unrecorded alcohol, which for most participants could happen 24 h a day. Illegal night sales of non-beverage alcohol (and in some cases samogon) from private apartments were reported for both cities, while legal sale of medicinal compounds from 24-h pharmacies was reported only in Barnaul, and illegal night sales of colognes only in Petrozavodsk.

Interview_B14_6 (male, 33 old): *“At 9PM nothing is sold anymore. But pharmacies are open 24-hours. So if a person didn’t manage to buy it until 9PM, but they want it [gestures] to the pharmacy. And pharmacies are nowadays at every corner, so at every corner there are -- So one can go there at night, anytime. Nearby my house, at walking distance, there are three pharmacies.”*

However, unsanctioned night sale of recorded alcoholic beverages was also reported in both cities.

### Presence of counterfeit alcohol in stores

Counterfeit alcohol manufactured on an industrial scale was reported to be very common in both cities, but especially widespread in Petrozavodsk and its surrounding areas. The majority of participants were very accustomed to alcohol counterfeits, frequently mentioning that counterfeiting is a common phenomenon in Russia, not limited to alcoholic beverages. Some interviewees expressed suspicion that the shadow economy of counterfeiting was ignored by state authorities, who did not enough to cease the systematic presence of counterfeit alcohol in retail sale and might even deliberately tolerate it because of corruption.

Interview_P14_5 (male, 47 years old): *“For the government, the main thing is: money. They keep repeating: corruption. But how do they fight corruption? They don’t fight any corruption there. The only thing they care about is how to put money in their own pockets. [ …]*. *They show it on TV: they’ve confiscated so and so many liters of alcoholic illegal products. But where is it going to? Like they say and show that they’re pouring it down the drain. But in reality, this is not true. The very same government passes this illegal alcoholic production over to the factories and puts it on the conveyers, delivers it to the stores and puts it on sale. They take it away from the underground organization and put it into their own industry.”*

Two types of counterfeiting were reported 1) non-deceptive counterfeits: a purchase scenario where it is reasonably foreseeable for the client that the obtained product is a counterfeit because of its low price, and 2) deceptive counterfeits, where the consumer cannot know for sure whether a counterfeit or an original product is purchased. While obvious counterfeiting was mainly reported for cheap alcopops and wines, blur counterfeiting occurred predominantly in the product range of spirits, most notably vodka. Interview participants could rarely distinguish between legally produced and declared vodka and illegally produced undeclared alcohol, which was bottled and sold as legal vodka.

Interview B14_7 (male, 33 years old): “*[And in the store, can you somehow guess, what is the normal vodka and what the counterfeit is?] No. I don’t think so. Some people rotate it, to see whether there is a twist or not, in the bottle. I don’t think that there is a difference at all. Nowadays they print it and make it in a way, that you could never tell the difference.”*

The professionalism and the complexity behind blur counterfeiting alcohol were emphasized. For instance, fake excise stamps and product labels were described as very sophisticated, without any visual differences from the original.

The only mentioned option to safely avoid consuming counterfeits was substituting homemade alcohol for regular alcoholic beverages.

### Changing status of artisanal alcoholic beverages in Russia

Unrecorded alcohol was found to be an initial commodity of the economic micro-cosmos of Russian communities. In its socio-economic sense, it fulfilled different functions for different individuals and was reported for the Russian cities as well as for the countryside. More artisanal beverages, primarily samogon, were reported for the village setting where it was available at night due to the limited opening hours of the local shops. Moreover, some village shops would offer only a very limited range of alcoholic beverages or no alcohol at all, since the official alcohol prices were too high for rural dwellers given their low salaries and the general level of unemployment. The smaller and more intimate communities allowed for easier circulation of recorded and unrecorded alcohol, as frequent money borrowing and semi-formal credit granting by local alcohol sellers were reported.

Participants also described how consumers would exchange information on local producers of unrecorded, who on their part would adapt their product range to the individual needs of their clients, for instance by selling or giving their high quality products to close relatives and friends, with whom they wanted to keep a good relationship, while selling low quality products to local heavy drinkers known for their alcohol dependence, from whom no complaints could be expected. Interviewees explained that these social relations put isolated alcohol dependent people at greater risk of obtaining unrecorded alcohol with certain by-products and potentially hazardous additives like sedatives or tobacco.

Generally, samogon was found to be a preferred product: depending on the context, it was described as a, cheap round-the-clock available alcoholic product, a “fluid currency” and object of economic exchange, a crucially important source of income or as a form of side business or, in some cases, a newly-discovered artisanal beverage increasingly gaining the status of a hand-made, trendy and yet traditional product.

Interview B14_5 (female, 45 years old): *“Our relatives come to visit us, they make [samogon] themselves. [...]They are generally abstainers, but [ …]* *this is their self-made. They cultivate the garden, it’s in the village.[...]So they come to visit us not empty-handed. To set up a good table for us.”*

Samogon was frequently described as a “natural” or “ecological” product, which would differ from the “chemical” and “artificial” beverages “with additives” one would find in the stores and very different than other unrecorded products. Accordingly, the countryside setting was often named as a more ecological environment, where samogon is predominantly produced. Also, a sharp boundary was clearly drawn between artisanal beverages and shop-bought alcohol, when it came to discussing the different effects of alcohol on the body. The majority of those interviewed emphasized that samogon would bear the lowest risk of alcohol associated harm compared to other kinds of alcoholic beverages.

Interview B14_1 (male, 42 years old): *“You cannot poison yourself with [samogon] and you will know for sure, that this is for real a natural product and not the counterfeit from the store. And[ …]* *it’s cheaper. [ … ]I won’t go to the store for the counterfeit, I would rather buy samogon and know for sure, that this person is producing a high quality product and it is 50 rubles cheaper. [The stores are selling counterfeits?] It happens. So especially … those brands.[...]The cheapest ones.[...]You can chance upon it in the stores really easily.”*

Moreover, the intimate atmosphere was pictured as a security mechanism, protecting the locals from obtaining low quality alcohol, especially counterfeits from regular retail sale.

Interview_P14_4 (male, 36 years old): *“In any city of Karelia [ …]* *any kind of vodka is not pure. [ …]* *So natural stuff that people consume now is [ …]* *braga [a home-made fermented alcoholic beverage][ …]* *Also samogon. I think this product will be purer, than the one from the store. I think it’s thousand times better than to buy from the shop. I’d rather buy it. Of course, I won’t buy non-beverage alcohol mixed with vodka. I won’t do that, but I would rather buy and drink samogon that people made themselves. As from the shop, even if I had a million, I won’t buy it anyway from the shop. I assure you. It’s the same with those Martinis, to hell with that. They’ll stick a label on the bottle: ‘Martini’. But what is mixed there?”*

Some of the interviewees also suggested that the increased state control over samogon distilling and higher fines would only aggravate the situation, as samogon was the only cheap option of high or medium quality alcohol with the only remaining alternative after that being low quality surrogates and counterfeits, often associated with greater health risks.

### Shifting markets of unrecorded alcohol

One of the major themes in the interviews was the shifting nature of the unrecorded alcohol market, moving in parallel with the introduction of alcohol policy. Participants observed a substantial re-organization of unrecorded alcohol markets in their communities, which has taken place over the last 10–15 years. In the course of this development availability of homemade alcohol has overall decreased, and the use of non-beverage alcohol and other surrogates has increased.

Interview_B14_7 (male, 33 years old): *“Samogon is not sold at all. I didn’t even hear anything about it. It’s only non-beverage alcohol that is sold. Back in the days it was sold, when I was a kid.”*

For Barnaul, a strong decline in samogon sales (and partially also sales of non-beverage alcohol from private apartments) was described for the city setting, while rural home-distilling was reported to be still common. Former producers and sellers of samogon were reported to switch to the less work-intensive sale of non-beverage alcohol or quit selling completely. The declining sales of homemade beverages were mainly explained by the smaller profit margins for the producers, compared to what they could earn with sales of non-beverage or smuggled alcohol.

Interview_B14_1(male, 42 years old): *“In the past, we used to produce [samogon] in the [district]. But now they do not do it anymore. [ …]* *Some produced it for their own use, some for selling. Even a lot produced it for selling. And now it’s easier to buy that non-beverage alcohol for 90 rubles and to sell it for 130.”*

For Petrozavodsk, interviewees reported the alcohol sales from apartments continued, but became now specialized on night sales of both, recorded and unrecorded alcohol, due to the introduced night ban on alcohol sale.

Besides economic factors, law enforcement was also mentioned as an important reason for the observed changes. In both cities, sales of homemade beverages and non-beverage alcohol had disappeared from the public view, but partly continued to take place in private houses and apartments, especially in the countryside, where sellers would sell their products only to clients they know, fearing legal punishment. Fines were named as the main measure and some participants mentioned imprisonment.

Interview_P14_3: (male, 34 years old): “*[In order to buy non-beverage alcohol] you have to send someone [the sellers would know personally]. Back in the days, I used to know these spots. But many of them were shut down. Many people got to jail.”*

Several previously very common and popular alcohol surrogates were reported to be no longer available; for instance, the “Troyar - vitalizing bath essence”, containing 90% ethyl alcohol, or the grain neutral spirit “Royal”, containing 96% ethyl alcohol, which was marketed as a cleaning agent in the 1990s. Consumers would then look for alternatives, comparable in price and availability, often switching to cosmetic and medicinal alcohols as a result.

One participant described the emergence of flavored antiseptic lotions on the legal market, which he felt were obviously produced for human consumption rather than for the declared purpose of skin disinfection.

Interview_B13_6 (male, 33 years old): *“It was sold in pharmacies and then they’ve began to produce it extra. Because it is made for injections, like for rubbing the skin before injection, so they started to produce it with cherry flavor, cranberries flavor, all different things and so forth. Then, they already forbid to sell it. They made it for the taste. Every pharmacy was selling it. The guys came with cars and were buying boxes full of it. They still drink it.”*

Since the number of individual sellers and producers of unrecorded alcohol has decreased, consumers of unrecorded products seem now to increasingly turn to cosmetic and medicinal alcohols, which are sold legally in pharmacies, kiosks and shops.

Interview_B13_10 (female, 29 years old): *“Fifteen years ago, at the street corner of my house [ …]* *old women [ …] were selling sun flower seeds and in the cartons they always had non-beverage alcohol. [ …] And now I know that this thing simply doesn’t exist anymore. Nowadays these [antiseptics] are the real deal. They’re bringing them here like I don’t know what. [So nowadays it has somehow moved to the pharmacies?] Yes, to the pharmacies. [ …]* *So earlier the people made the money and nowadays it’s the state, it seems. As it seems to be profitable.”* 11.40–12.04.

### Consumer’s views on alcohol policies

Interviewees voiced a general mistrust towards the recently implemented alcohol regulations, and expressed particular concerns over price increases and restriction of night sales of alcoholic beverages. From the consumer’s point of view, the sale of unrecorded alcohol has continued and some participants pointed out that unrecorded consumption was increasing in their communities because of growing prices for regular alcohol and sale time restrictions.

Interview_B14_7 (male, 33 years old): *“Nowadays it’s more about what’s sold from apartments. The stuff from the shops is less consumed. People mainly buy the things from apartments. Because now they made it like it’s only until 9 PM. But what should people do after 9 PM?*

Price increases affected individuals differentially, depending on their socio-economic status and their drinking behaviors; while affluent individuals would go to bars and restaurants to purchase recorded beverages, poorer members of the community would have to turn to different venues to buy cheap unrecorded alcohol illegally.

Interview_P14_2 (female, 37 years old): *“Back in the days it was easier with alcohol. It was sold 24-hours.[ …]*. *People were buying it more from the stores. And now, people have only limited possibilities. And that’s why it seems to me, that people started to consume more of those [unrecorded] drinks. Because there is no other possibility. Not everyone is rich. Not everyone would go to a bar and a restaurant and buy a bottle of vodka, for example, which would cost there more than 1000 rubles. So maybe it’s better to go to acquaintances and to buy that samogon for 100 and something rubles? Or a bottle of vodka for 200 rubles”.*

Sales of both recorded and unrecorded alcoholic products were reported for different selling spots, ranging from legal purchasing scenarios in pharmacies, to illicit sales of alcoholic beverages in shops, and to illegal sales of unrecorded alcohol in private apartments, houses, kiosks or taxis. The newly formed informal night market adapted flexibly to the needs of the various consumer groups, their purchasing power and product preferences, and local sellers found inventive ways to evade the new laws. For instance one participant described how a shop would be presented as a bar in order to sell alcohol at night time or how the minimum price law on vodka would be violated through special offers.

Interview_B14_4 (male, 36 years old): *“They allowed selling [alcohol] after 9 PM only [ …]* *in bar places, where people are staying and drinking. So take-aways are forbidden. So they immediately [ …]* *mounted [a table] at the wall as a bar counter and started to sell at night. [ …]* *A [minimal price] law about vodka was passed that vodka must cost not less than 180 rubles. [ …]* *I go to the shop and there is that vodka standing, the cheapest one, the shittiest one. There is a small 0.25liter bottle attached to it with duct tape and a sign ‘a present’. For 180 rubles. So for the money, for the same money you’re buying ‘a present’. They just taped it down over night, wrote the signs and: ‘That’s the law. We don’t violate it.’ These laws are -- if you’re forbidding it, you have to forbid it for real. And this way it’s – you just know all these loopholes.”*

Although public consumption of alcohol was officially restricted, participants noted that it has not totally disappeared from the Russian streetscape. Individuals and groups would still consume alcohol in the streets, but now in a more discrete way, for instance, in the courtyards rather than more visible spots.

Despite reporting a decline in sales outlets of unrecorded alcohol from private individuals, interviewees saw no overall decline in alcohol consumption. When asked whether any changes were observed over the last 10–15 years, the majority of interviewees stated that they either observed no changes (i.e., that high alcohol consumption and alcohol-related mortality remained one of the main problems of their communities, along with general poverty and low living standards), or that the situation was getting worse (i.e., that the huge shadow market of alcohol counterfeits, as well as legal sales of cosmetic and medicinal alcohols, were resulting in an increase in consumption of unrecorded products).

## Discussion

### Principal findings

The overall findings confirmed previous studies that unrecorded alcohol consumption is common in the population of patients, who are registered with AUDs in the Russian narcology system [3, 13, 19, 42, 43]. The results demonstrate that unrecorded alcohol plays a crucial role in the lived experiences of the study participants, shaping their communities in economic and social means and providing a cheap source of alcohol, despite the introduced alcohol control policies since 2005/2006.

Affordability and availability of recorded and unrecorded alcohol were described as key factors in shaping distinct drinking behaviors: some participants reported they would stop drinking once no more recorded alcohol could be bought, while others would purchase unrecorded products instead.

High availability of unrecorded alcohol appeared to be especially relevant for these consumers who fulfil the criteria for AUDs and have already developed problematic drinking patterns harmful to health; it is notable that they have also reached a certain level of social marginalization. While home-made alcohol was considered to be a good alternative to recorded alcohol and was viewed as a guarantee against counterfeit alcohol, surrogates were ascribed to poor, marginalized and severely dependent individuals and particularly homeless heavy drinkers. Moreover, consumption of surrogates was perceived as a social marker for marginalization – the severity level of someone’s alcohol dependence and social declassification was judged by whether a person consumed surrogates, and this view is supported by the consumers and non-consumers of unrecorded alcohol alike [23].

Surrogates were reported to be the cheapest and the most available type of alcoholic products, with medicinal compounds being especially accessible due to their legal status, the high numbers of pharmacies and their extensive opening hours. The qualitative accounts indicate that the implemented measures of denaturation and taxation of non-beverage alcohol and the increased penalties for unlicensed sale of homemade beverages seemed to have helped to decrease private sales of home-made and non-beverage alcohol, but many consumers might have switched to medicinal and cosmetic alcohols instead.

The Federal Law and the Tax Code of the Russian Federation allow for legal and unlicensed sale of these untaxed products, which has apparently resulted in a growing market of surrogates- alcoholic products deliberately designed for human consumption, but declared otherwise to avoid taxation [18, 31]. Descriptions of cosmetic lotions, which are officially promoted as antiseptics for external application only, but come with different flavors, corroborate this idea. Different than medicinal lotions, these products are sold not in pharmacies, but mainly in kiosks and small shops [18] and could also be purchased in bulk from various online shops at the time of the study [35].

From the participants’ perspective, the recently implemented measures of higher taxation of alcoholic beverages, restrictions of alcohol sale time and sale locations as well the ban on public drinking had little effect, since night sales of alcohol continued to take place in different forms. Moreover, the increasing prices of alcohol would push individuals with problematic drinking patterns into consuming unrecorded alcohol, once recorded beverages were no longer affordable. While the shifts from recorded to the unrecorded market seem to be wide-spread in the population of people with alcohol-dependence, the general population is the main target of the new policies. This study sample encompassed patients who were officially registered in a state-run treatment facility and diagnosed with alcohol dependence –a special population, since state-registered patients are more likely to be at the lower end of the socio-economic scale and to be socially marginalized. Research that involves patients from the narcological setting in Russia seems crucial for the assessment of consequences of alcohol control measures in a population that is among the most vulnerable to the very problems the policies are intended to address.

### Implications for alcohol policy

Despite the often voiced concern that price increases and sale restrictions of alcoholic beverages might encourage consumers to switch from recorded beverages to unrecorded products [16], no such tendencies were found in studies on the general population [10, 27, 28], albeit in studies limited in their inclusion of types of unrecorded alcohol.

The present findings suggest that structural changes of unrecorded alcohol markets have taken place in the course of the last decades, which had also an impact on the suppliers and sellers as well as consumers of unrecorded alcohol.

Although the number of individual manufacturers and sellers of unrecorded alcoholic products seems to have decreased over the last decade, their place was apparently taken by larger industrial producers, meeting the stable demand for cheap alcoholic products and sustaining unrecorded consumption in the communities. Two large contemporary suppliers of unrecorded alcohol were identified at the time of the study: 1) corporations legally selling and producing cosmetic and medicinal alcohols as surrogates and 2) the illegal counterfeit alcohol sector.

Descriptions of homemade beverages and most importantly samogon depict a general status change of artisanal beverages within the Russian society, in which they seem to become the beverage of choice for the broader population. This image change happens against the backdrop of the systematic presence of alcohol counterfeits in Russia – a problem obviously affecting not only the cheapest beverages but also high profile brands and hence more affluent consumers, which is in line with the existing research in the field [11, 19].

Therefore, the results point to the possibility that a substation of recorded alcohol for unrecorded products might have taken place at least in certain consumer groups, namely people with AUDs of lower socio-economic strata, after certain alcohol control measures on recorded alcohol were introduced. Price increases and limitations of alcohol availability (most importantly the nation-wide night ban on alcohol sale) seem to be the most influential policies in this regard. However, due to the small sample size of the study, which is also based on self-reports of a special population only, these findings should be interpreted with caution.

As indicated above, large-scale quantitative studies on the consumption of unrecorded in the general population do not exist or do not cover the entire spectrum of unrecorded alcohol products [27, 28]. Nonetheless, the existing estimates in the field that are either based either on strong correlations between alcohol-attributable mortality and total alcohol consumption ([15, 20]; Nemtsov et al., in press) or on expert assessment and modelling of available survey data [26] suggest that unrecorded consumption has decreased in Russia. The estimates provided by the WHO, which rely on a combination of the outlined approaches, indicate that unrecorded consumption has decreased by about 23% between 2003 and 2005 and 2016 [45, 46].

Existing theories on population consumption, most notably Skog’s theory of collective drinking behavior, postulate a a close relation between the mean consumption level in a given population and the prevalence of heavy drinkers. If alcohol consumption increases in the whole population, the number of individuals above a certain consumption limit will be increasing as well [38]. This notion has been influential also in informing and formulating alcohol policy as it allowed identifying entry points and target areas for delivering effective policy measures, namely the reduction of per capita alcohol consumption, prevalence of heavy drinking and availability of alcohol in a given population.

For Russia, as for most other countries, empirical data on the consumption trends in different population segments (including consumption of unrecorded alcohol) is lacking and according research is difficult to organize, which is why qualitative inquiries as presented in this contribution appear to be an important source of information on the changing drinking contexts and the implications of alcohol policy in different population groups.

The discussed findings suggest that pricing policies as an isolated measure might run the risk of increasing unrecorded alcohol consumption among individuals with AUD, who have reached a certain level of socio -economic marginalization, which is why additional measures are needed as part of a unified policy framework to address unrecorded alcohol [21, 22]. The policies introduced after the mentioned Irkutsk mass methanol poisoning, most importantly the sales ban on certain types of non-beverage alcoholic products and the harsher penalties on illicit alcohol production and sale, appear to be most promising measures in reducing unrecorded alcohol consumption in all population segments through a general reduction of availability. However, as these measures were adopted after the present study took place, they cannot be discussed as part of this contribution.

### Limitations

There are a number of limitations to our study. The anonymous interviews are prone to the usual biases of self-report studies, including social desirability or memory bias. The interviews were conducted in two very different regions of Russia that have their distinct features – Barnaul as a large deindustrialized city in West-Siberia, whereas Petrozavodsk is a smaller city in Karelia in the Russian North-West. Therefore the interviewee’s perceptions of changes within their communities are heavily influenced by the general socio-economic and political changes in their regions, which might have been more extreme in the case of the de-industrialization crisis in Barnaul and the Altai region in the 1990s and cannot be generalized for other regions. Thus, the interviews might explain only local phenomena in unrecorded consumption, especially when it comes to the perceived changes of the unrecorded alcohol markets. Moreover, the study was conducted in two different types of narcological institutions – the outpatient ambulatory centers (Barnaul) and inpatient clinics (Petrozavodsk). However, in analyzing the interviews, we could not see distinct differences by either region or type of service, which strengthens our interpretation of more general characteristics associated with unrecorded consumption in people with AUDs in Russia. Moreover, the current study was conducted before the Irkutsk mass methanol poisoning and the according legislations tackling the sale of non-beverage alcoholic products such as the antiseptic lotions. Hence, it is to expect that there were further shifts in the described market dynamics and newer accounts are needed to document this.

Finally, it should be acknowledged that marginalized consumers are not limited to AUD patients treated in state-run narcology services; a big proportion of individuals, who fulfil the clinical criteria of AUD, never reach out for help and are never treated. Given the outlined specificities of Russian treatment systems, this is most likely an even bigger proportion in Russia as compared to other countries with differing treatment systems. Therefore, the present study might have overlooked the perceptions of other marginalized (and potentially larger) consumer groups as they were not reached by the sampling procedure.

## Conclusion

In spite of a number of new alcohol policies introduced in the course of the past several years, consumption of unrecorded alcohol remains of public health concern in Russia. Our in-depth interview study describes specific drinking patterns of unrecorded alcohol and highlights how people with alcohol dependence, many of whom are consumers of unrecorded alcohol, perceive different alcohol control measures. Their first-hand accounts indicate that the policy measures undertaken might have led to a substantial shift in unrecorded alcohol markets, and influenced purchase behaviors of heavy drinkers, who are the main consumers of unrecorded alcohol. Moreover, at least some of the measures – first and foremost price increases – might have led to negative consequences as recorded alcohol became increasingly unaffordable for individuals from lower socio- economic strata. Combined with the ubiquitous presence of different sources of cheap unrecorded alcohol, such policies might marginalize disadvantaged population groups even more.

To tackle the issue of mortality and harm arising from unrecorded consumption as well as to prevent health inequalities from widening, alcohol control policies should systematically address unrecorded alcohol and aim towards the overall reduction in its availability. Although alcohol control measures cannot reach every consumer and lead to according behavior change as various barriers exist, including personal ones such as lack of motivation or insight to alter behavior, their effectiveness and need from a public health perspective remains unquestioned.

International experience shows that alcohol policies need to target various areas at the same time in order to be effective and to avoid unintended and unwanted consequences – according measures have to aim at the alcohol producers and the entire alcohol supply chain, the drinking environment, the health system as well as the individual drinkers [1].

As the results suggest that the existing alcohol control measures are mostly population-based and seem to fail to reach the marginalized sub-groups of unrecorded alcohol consumers, specific high-risk strategies appear to be needed to reduce harm from unrecorded consumption in Russia. The implementation of screening and brief interventions for excessive alcohol consumption in various contexts such as primary healthcare settings, trauma treatment services or the workplace could be such a strategy.

## Supplementary information

**Additional file 1: Table S1.** Sociodemographic characteristics of the study sites. **Table S2.** Prevalence and incidence of alcoholic psychoses and alcohol dependence for the researched regions. **Table S3.** *Sample characteristics.* **Figure S1**. Full thematic map of the overall interview study (for the sub-study on unrecorded drinking patterns and harm see: [23]).

## Data Availability

The dataset is not publicly accessible, but available from the corresponding author on a request.

## Abbreviations

AUD: Alcohol use disorder
REB: Research Ethics Board
WHO: World Health Organization
