# Supplementary material for: Perception of alcohol policies by consumers of unrecorded alcohol - an exploratory qualitative interview study with patients of alcohol treatment facilities in Russia
Source: Subst Abuse Treat Prev Policy. 2019 Nov 21;14:53. doi: 10.1186/s13011-019-0234-1 (PMC6869256; doi:10.1186/s13011-019-0234-1)
Supplement: Supplementary file 1 — Additional file 1: Table S1. Sociodemographic characteristics of the study sites. Table S2. Prevalence and incidence of alcoholic psychoses and alcohol dependence for the researched regions. Table S3. Sample characteristics. Figure S1. Full thematic map of the overall interview study (for the sub-study on unrecorded drinking patterns and harm see: [23]). [file 13011_2019_234_MOESM1_ESM.docx]

# WEBAPPENDIX A

Further information on the sample and the study sites.

Table W1: Sociodemographic characteristics of the study sites

| **Characteristics** | **Altai Krai** | **Republic of Karelia** |
| --- | --- | --- |
| Total Population (in 2014) | 2,390,638 (1.66% of total Russian population)  Barnaul (capital): 632,848 | 634,402 (0.44% of total Russian population)  Petrozavodsk(capital): 272,101 |
| Urban-rural population ratio (in 2014) | 55.76% urban, 44.24% rural | 79.18% urban,  20.82% rural |
| Death rate (in 2013) | 14,2 (per 1,000) | 14.7 (per 1,000) |
| Population decline (in 2013) | - 0.7% | - 2.7% |
| Ranking position of life quality ranking of Russian regions in 2014 (assessment of life quality was based on aggregated data) | 64 (out of 82) | 65 (out of 82) |
|  | **Barnaul** (capital) | **Petrozavodsk** (capital) |
| Living wage per capita and month | 6,565 RUB (2013)  7,423 RUB (2014) | 8,324 RUB (2013)  9,319 RUB (2014) |
| Average wage per capita and month | 14,752 RUB (2013)  17,134 RUB (2014) | 21,494 RUB (2013)  22,939 RUB (2014) |
| Percentage of people living below living wage | 17.6% (2013)  17.0% (2014) | 14.1% (2013)  14.2% (2013) |
|  | **Market value of the Russian ruble for the assessment periods** | |
| August 2013 | 1 RUB ≈ 0.023 € ≈ 0.03 $ | |
| August 2014 | 1 RUB ≈ 0.021 € ≈ 0.028 $ | |
| September 2014 | 1 RUB ≈ 0.02 € ≈ 0.026$ | |
|  | **Alcohol and foodstuff prices** **for Russia** (third quarter of 2014)* | |
| Vodka (40% ethanol, 0,5l) | 220-250 RUB (≈ 6.16- 7.0$) with an established fixed minimum price of 220 RUB (≈ 6.16$). | |
| Beer (4-6% ethanol, 0,5l) | 40-50 RUB (≈ 1.12-1.4$). | |
| Home-distilled samogon (0,5l, 50-80% ethanol) | 80-150 RUB (≈ 2.24-4.2$). | |
| Medicinal tincture and antiseptic (100ml, 70% and 95% ethanol, respectively) | 15-25 RUB (≈ 0.42-0.70$) | |
| Eau de cologne (86ml, 60-% ethanol) | 20 RUB (≈ 0.56$). | |
| Bread (0,5kg) | 20-30 RUB (≈ 0.56-0.84$) | |
| Milk | 40-60 RUB (≈ 1.12-1.68$) | |

Table W2: Prevalence and incidence of alcoholic psychoses and alcohol dependence for the researched regions.

|  | **Prevalence of alcoholic psychoses** | | | |
| --- | --- | --- | --- | --- |
|  | Absolute number | | Per 100,000 | |
|  | **2013** | **2014** | **2013** | **2014** |
| Russian Federation | 75,756 | 72,180 | 52.79 | 49.41 |
| Republic of Karelia | 619 | 789 | 97.38 | 124.55 |
| Altai Region | 731 | 842 | 30.53 | 35.26 |
|  | **Prevalence of alcohol dependence (incl. alcoholic psychoses)** | | | |
|  | Absolute number | | Per 100,000 | |
|  | **2013** | **2014** | **2013** | **2014** |
| Russian Federation | 1,852,598 | 1,809,975 | 1290.95 | 1238.94 |
| Republic of Karelia | 7,078 | 7,750 | 1113.48 | 1223.42 |
| Altai Region | 36,251 | 34,560 | 1513.81 | 1447.40 |
|  | **Incidence of alcoholic psychoses** | | | |
|  | Absolute number | | Per 100,000 | |
|  | **2013** | **2014** | **2013** | **2014** |
| Russian Federation | 33,483 | 3,2056 | 23.33 | 21.94 |
| Republic of Karelia | 304 | 345 | 47.82 | 54.46 |
| Altai Region | 667 | 809 | 27.85 | 33.88 |
|  | **Incidence of alcohol dependence (incl. alcoholic psychoses)** | | | |
|  | Absolute number | | Per 100,000 | |
|  | **2013** | **2014** | **2013** | **2014** |
| Russian Federation | 112,218 | 10,9149 | 78.20 | 74.71 |
| Republic of Karelia | 812 | 909 | 127.74 | 143.50 |
| Altai Region | 2,518 | 2,669 | 105.15 | 111.78 |

*Table W3: Sample characteristics*

| **Sample characteristics** | **Barnaul (2013 and 2014)** | **Petrozavodsk (2014)** | **Total Sample Description** |
| --- | --- | --- | --- |
| **Sample size** (n) | 18 | 7 | 25 |
| **Age** (in years, mean, SD and range) | M: 41.33 SD; SD: 13.54; range 29-78 | M: 39.14; SD: 11.9; range: 24 - 60 | M: 38.72; SD: 15; range: 24 - 78 |
| **Female gender** (in % of total sample size) | 94.4% (7) | 14.4% (1) | 32%(8) |
| **Place of residence** |  |  |  |
| Urban | 94.4% (17) | 85.7% (6) | 92% (23) |
| Rural | 4.4% (1) | 14.3% (1) | 8% (2) |
| **Education**  (in % of total sample size) |  |  |  |
| Primary school education, basic vocational training or less | 33.3%(6) | 71.4% (5) | 44% (11) |
| Secondary/ Secondary specialized | 50% (9) | 28.6% (2) | 44% (11) |
| Higher Education | 16.7% (3) | 0 | 12% (3) |
| **Employment**  (in % of sample size) |  |  |  |
| Unemployed | 38.9% (7) | 28.6% (2) | 36% (9) |
| Informal/occasional employment (unskilled labor) | 22.2% (4) | 42.9% (3) | 28%(7) |
| Formal employment (skilled labor) | 16.7% (3) | 14.3% (1) | 16% (4) |
| Self-employment | 11.1% (2) | 0 | 8% (2) |
| Retired | 11.1% (2) | 14.3% (1) | 12% (3) |
| Periods of unemployment reported | 61.1% (11) | 71.4% (5) | 64% (16) |
| Imprisonment reported | 18.2% (2) | 0 | 8% (2) |

# WEBAPPENDIX B

Thematic maps of analyzed material.


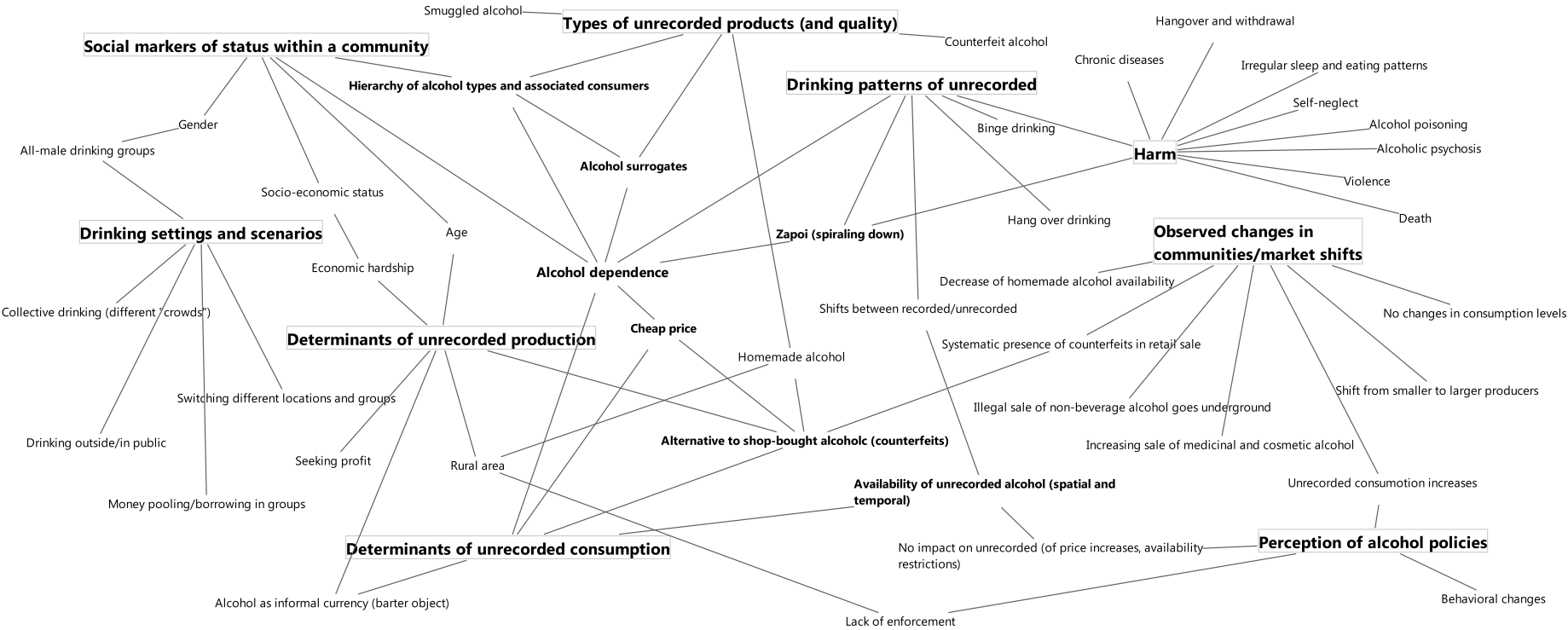


Figure W1. Full thematic map of the overall interview study (for the sub-study on unrecorded drinking patterns and harm see: Neufeld et al., 2017a).
